# An improved method for the heterologous production of soluble human ribosomal proteins in *Escherichia coli*

**DOI:** 10.1038/s41598-019-45323-8

**Published:** 2019-06-20

**Authors:** Danilo Correddu, José de Jesús Montaño López, Praveen G. Vadakkedath, Amy Lai, Jane I. Pernes, Paris R. Watson, Ivanhoe K. H. Leung

**Affiliations:** 1 School of Chemical Sciences, The University of Auckland, Private Bag 92019, Victoria Street West, Auckland, 1142 New Zealand; 2 Facultad de Ingeniería, Universidad Nacional Autónoma de México, Av. Universidad 3000, Ciudad Universitaria, Coyoacán, Cd. Mx. CP 04510 Mexico; 3 The MacDiarmid Institute for Advanced Materials and Nanotechnology, Victoria University of Wellington, PO Box 600, Wellington, 6140 New Zealand; 4 School of Cellular and Molecular Medicine, University of Bristol, Biomedical Sciences Building, University Walk, Bristol, BS8 1TD United Kingdom; 5 School of Biological Sciences, Victoria University of Wellington, PO Box 600, Wellington, 6140 New Zealand; 6 Maurice Wilkins Centre for Molecular Biodiscovery, The University of Auckland, Private Bag 92019, Victoria Street West, Auckland, 1142 New Zealand

**Keywords:** Biological techniques, Chemical biology

## Abstract

Human ribosomal proteins play important structural and functional roles in the ribosome and in protein synthesis. An efficient method to recombinantly produce and purify these proteins would enable their full characterisation. However, the production of human ribosomal proteins can be challenging. The only published method about the recombinant production of human ribosomal proteins involved the recovery of proteins from inclusion bodies, a process that is tedious and may lead to significant loss of yield. Herein, we explored the use of different *Escherichia coli* competent cells and fusion protein tags for the recombinant production of human ribosomal proteins. We found that, by using thioredoxin as a fusion protein, soluble ribosomal protein could be obtained directly from cell lysates, thus leading to an improved method to recombinantly produce these proteins.

## Introduction

The human ribosome is a complex bio-machinery that is responsible for protein biosynthesis. It is formed by four molecules of RNA and 82 proteins. Ribosomal proteins are involved in many important cellular processes. They play a role in the maturation of rRNA and ribosome, they act as structural and functional components of the ribosome^1^, and they are involved in physiological processes like the activation of the tumour protein p53^2–4^. Mutations in ribosomal proteins are often found to be directly connected to different types of diseases, thus ribosomal proteins are potential inhibition targets in drug development^5–7^.

Recently, several high-resolution structures of the ribosome have become available by using X-ray crystallography and cryo-electron microscopy^8–12^. These structures reveal important (atomic) details about the three-dimensional arrangement of its components. However, whilst these structures allow a global understanding about the ribosomal machinery, specific information about individual ribosomal proteins, for example protein dynamics, protein-protein or protein-RNA interactions, are difficult to obtain directly from these structures.

To date, only a handful of human ribosomal protein structures have been characterised. Information about the function and intermolecular interactions of individual human ribosomal proteins are often obtained using homologues from prokaryotes or yeast^13^. However, some eukaryotic ribosomal proteins do not have prokaryotic homologues. It is also known that the biological functions of some human proteins are different from their bacterial versions^14^. An efficient way to produce recombinant human ribosomal proteins would therefore allow these proteins to be studied. However, ribosomal proteins are basic molecules, as reflected by the large number of arginine and lysine residues in their primary structure when compared to non-ribosomal proteins^15,16^. Thus, their recombinant production and purification can be challenging. In fact, the only published method about the production of recombinant human ribosomal proteins involves recovering the protein from inclusion bodies, a process that is tedious and may lead to significant loss of yield^17^.

Here, we explored different strategies to improve the heterologous production of human ribosome proteins by using the *Escherichia coli* expression system. We found a simple and efficient method for the expression and purification of soluble human ribosomal proteins by using thioredoxin as a fusion protein, which improves solubility of the ribosomal protein, and helps avoid the accumulation of recombinant proteins into the bacterial inclusion bodies. As the gene sequences of human ribosomal proteins typically contain rare codons that could slow down or impede the regular mRNA translation process in *Escherichia coli*^18^, the use of *E. coli* competent cells that contain a higher level of tRNAs that recognise rare codons were also explored.

## Results and Discussion

### Selection of human ribosomal proteins

Our goals of this work were to find a method that allows us to obtain soluble human ribosomal proteins directly from cell lysates and avoids the inconvenient procedure of protein recovery from inclusion bodies. We also wanted to understand the factors that affect the stability and solubility of ribosomal proteins. In order to minimise the variables in this study, we chose four human ribosomal proteins that are of similar size, isoelectric point and amino acid composition; They are S10, S15, S18 and L11 (Tables 1 and 2).

**Table 1:** Ribosomal proteins characteristics: S10, S15, S18 and L11.

	S10	S15	S18	L11
Molecular Weight	18897.77 Da	17040.1 Da	17718.68 Da	20252.39 Da
Isoelectric Point	10.15	10.39	10.99	9.64

**Table 2:** Ribosomal proteins amino acid composition and their percentage of total amino acids.

	S10		S15		S18		L11	
ALANINE (A)	14	8.5%	5	3.4%	8	5.3%	10	5.6%
ARGININE (R)	18	10.9%	15	10.3%	20	13.2%	17	9.6%
ASPARAGINE (N)	4	2.4%	3	2.1%	6	3.9%	6	3.4%
ASPARTIC ACID (D)	6	3.6%	4	2.8%	9	5.9%	8	4.5%
CYSTEINE (C)	0	0.0%	0	0.0%	0	0.0%	4	2.2%
GLUTAMINE (Q)	6	3.6%	7	4.8%	6	3.9%	7	3.9%
GLUTAMIC ACID (E)	12	7.3%	10	6.9%	7	4.6%	14	7.9%
GLYCINE (G)	15	9.1%	10	6.9%	12	7.9%	19	10.7%
HISTIDINE (H)	5	3.0%	5	3.4%	5	3.3%	3	1.7%
ISOLEUCINE (I)	4	2.4%	7	4.8%	11	7.2%	15	8.4%
LEUCINE (L)	13	7.9%	14	9.7%	14	9.2%	13	7.3%
LYSINE (K)	13	7.9%	16	11.0%	16	10.5%	16	9.0%
METHIONINE (M)	6	3.6%	8	5.5%	2	1.3%	3	1.7%
PHENYLALANINE (F)	6	3.6%	5	3.4%	4	2.6%	8	4.5%
PROLINE (P)	15	9.1%	8	5.5%	3	2.0%	5	2.8%
SERINE (S)	5	3.0%	7	4.8%	3	2.0%	7	3.9%
THREONINE (T)	6	3.6%	6	4.1%	9	5.9%	7	3.9%
TRYPTOPHAN (W)	2	1.2%	0	0.0%	2	1.3%	1	0.6%
TYROSINE (Y)	7	4.2%	6	4.1%	3	2.0%	6	3.4%
VALINE (V)	8	4.8%	9	6.2%	12	7.9%	9	5.1%
TOTAL AMINO ACIDS	165		145		152		178	

The recombinant production of S15 and S18 has not been reported to date. The recombinant production of S10 has been reported^17^. Its production required the use of denaturing agents to solubilise the protein from inclusion bodies. This was then followed by refolding so that the native structure of the protein can be obtained. However, such method is time-costly. It is also inefficient as the rate of recovery can be slow, with enzymes losing their activity as they return to their original folded state^19^. The production of L11 has also been reported^3^. It was found that soluble GST-tagged L11 could be obtained if it was co-expressed and co-purified with mouse double minute 2 (MDM2), a protein that was known to form a stable complex with L11. However, in the absence of MDM2, L11 rapidly precipitated at low concentrations^2^.

### Recombinant production of human ribosomal proteins with poly-histidine tag

Our first approach was to produce soluble proteins with a N-terminal poly-histidine tag for affinity chromatography purification and compare it with the refolding protocol that was reported by Malygin *et al*.^17^. The gene sequences of S10, S15, S18 and L11 were first cloned into the expression vector pNIC28-Bsa4. This vector was designed for high-throughput production of human proteins^20^. It enables the production of recombinant proteins with a N-terminal poly-histidine tag and a tobacco etch virus (TEV) protease cleavage site. The resulting plasmids were introduced into *E. coli* BL21 (DE3) competent cells and protein expression trials were conducted to find the optimal growth condition to obtain soluble ribosomal proteins from the cell lysate. Several tests were carried out by varying the temperature and time of incubation (18 to 37 °C; 4 hours to overnight), and concentration of isopropyl β-D-1-thiogalactopyranoside (IPTG) for the induction of protein expression (0, 0.1 and 1 mM).

**Figure 1:**
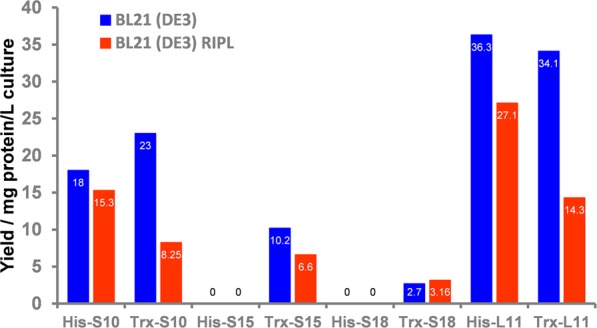
Production of soluble ribosomal proteins from the cell lysate expressed in mg per litre of culture. Poly-histidine-tagged recombinant proteins are indicated as His-S10, His-S15, His-S18 and His-L11. Recombinant proteins fused with a poly-histidine-thioredoxin tag are indicated as Trx-S10, Trx-S15, Trx-S18 and Trx-L11. When expressed in *E. coli* BL21 (DE3), thioredoxin improves the total production of S10, S15 and S18 (in blue). When the genes are expressed in *E. coli* BL21 (DE3) CodonPlus RIPL (red), the total production of soluble protein is lower compared to *E. coli* BL21 (DE3).

**Figure 2:**
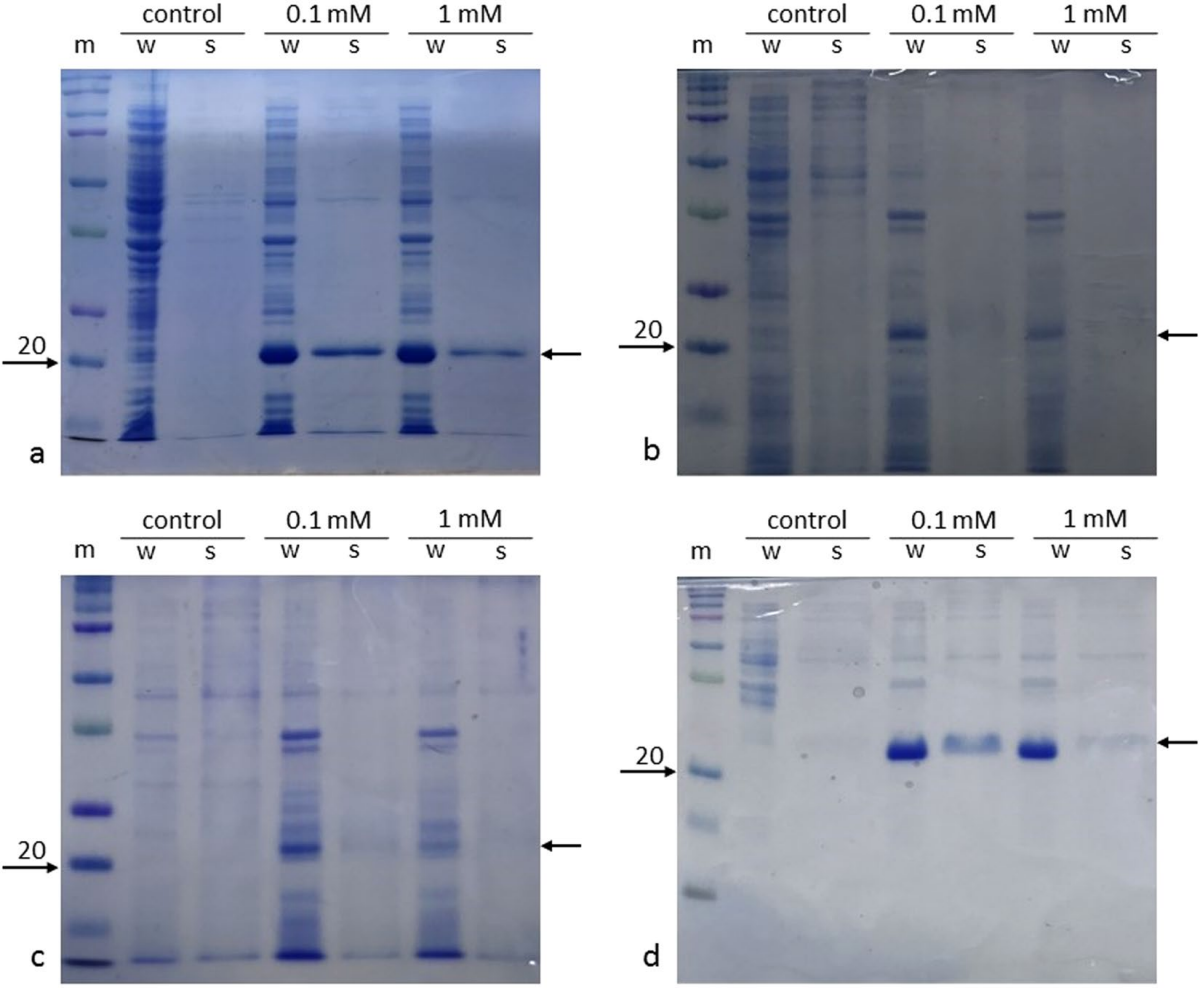
SDS-PAGE analysis of protein expression trials in *E. coli* BL21 (DE3) using pNIC28-Bsa4. The temperature was 18 °C. Lanes legend: m = molecular weight protein marker; w = whole cell sample; s = soluble proteins. Every gel includes non-induced samples (control), and samples induced with 0.1 mM and 1 mM IPTG. Gels show expression trials of the poly-histidine-tagged ribosomal proteins S10 (**a**), S15 (**b**), S18 (**c**) and L11 (**d**). Arrows on the right side of the gels indicate the expected positions of poly-histidine-tagged proteins. Soluble His-S10 and His-L11 (21.6 and 22.9 kDa) are more abundant in samples induced with 0.1 mM IPTG. No bands are present at the expected molecular weights for ribosomal proteins S15 and S18. Photos of the SDS-PAGE gels were taken and cropped using the mobile application Microsoft OneNote for iPhone. No adjustments in colour or contrast were made. Full-length SDS-PAGE gels are provided in the Supplementary Fig. S13.

Interestingly, in contrast to previously published reports^17^, we found that some ribosomal proteins (S10 and L11) could be produced and purified in high quantities directly from cell lysates when the genes were expressed at 18 °C overnight and induced with 0.1 mM IPTG (Figs 1 and 2, and Supplementary Figs S1 and S2). The amount of soluble S10 produced in this work (~18 mg) per litre of culture was about 2/3 of the amount of S10 that was recovered from inclusion bodies, as described by Malygin *et al*. (~28 mg; Fig. 1)^17^. On the contrary, the soluble production of S15 and S18 were unsuccessful amongst all the tested conditions (Fig. 2). Our results suggest that the four selected human ribosomal proteins have different levels of expression and solubility despite their similarity in size and composition. For proteins such as S10 and L11, denaturation and refolding are not required as it is possible to purify them in high quantity directly from the soluble fraction. We also found that, as a general rule-of-thumb, the formation of insoluble proteins in the inclusion bodies can be contained by growing the cultures at lower temperatures and by using a low concentration of IPTG.

### Thioredoxin improves the solubility of unstable proteins

**Figure 3:**
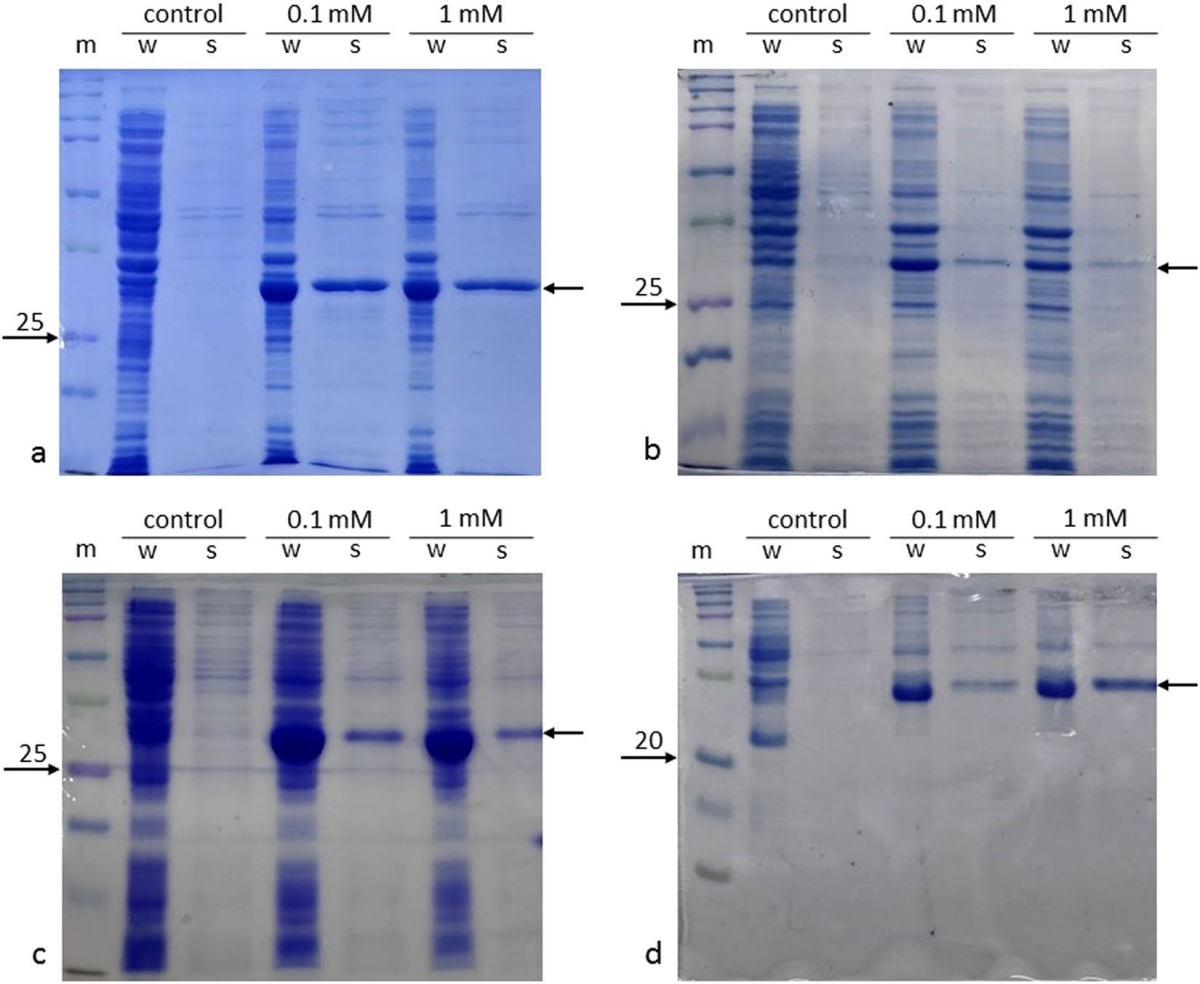
SDS-PAGE analysis of protein expression trials in *E. coli* BL21 (DE3) using pNH-TrxT. The temperature was 18 °C. Lanes legend: m = molecular weight protein marker; w = whole cell sample; s = soluble proteins. Every gel includes non-induced samples (control), and samples induced with 0.1 mM and 1 mM IPTG. Gels show expression trial of the poly-histidine-thioredoxin-tagged ribosomal proteins S10 (**a**), S15 (**b**), S18 (**c**) and L11 (**d**) Arrows on the right side of the gels indicate the expected positions of recombinant proteins. Soluble Trx-S10 and Trx-S15 and Trx-S18 (33.1, 31.2 and 31.9 kDa) are more abundant in samples induced with 0.1 mM IPTG. Trx-L11 (34.4 kDa) is highly expressed when induced with 1 mM IPTG. Photos of the SDS-PAGE gels were taken and cropped using the mobile application Microsoft OneNote for iPhone. No adjustments in colour or contrast were made. Full-length SDS-PAGE gels are provided in the Supplementary Figure S14.

As the use of the pNIC28-Bsa4 vector did not allow the recovery of S15 and S18 directly from cell lysates, we therefore decided to investigate the use of additional fusion tags to help enhance the solubility of recombinant proteins^21^. According to Hammarstrom *et al*.^22^, thioredoxin is amongst the best fusion proteins to improve the solubility of small human proteins in *E. coli*. Many expression vectors have been designed and used to express heterologous genes, including pNH-TrxT, which is a derivate of pNIC28-Bsa4. This vector enables the production of recombinant proteins together with a N-terminal poly-histidine-thioredoxin tag and TEV protease cleavage site. We decided to clone the four genes (that encode S10, S15, S18 and L11) into pNH-TrxT and compare the soluble production of the poly-histidine-thioredoxin-fused ribosomal proteins with the poly-histidine-tagged proteins produced using the expression vector pNIC28-Bsa4 (Supplementary Figs S3–S6). Surprisingly, we found that the amount of soluble S10 and L11 were similar to the amount of protein produced with the sole poly-histidine tag (Fig. 1). For large-scale production of L11, the amount of protein produced increased three fold when the gene expression was induced with 1 mM IPTG, compared to the production achieved with 0.1 mM. In order to avoid rapid aggregation and precipitation of L11, the elution needed to be diluted and frozen with liquid nitrogen promptly before storage at −80 °C. Under these conditions, S15 and S18 could be produced and purified from cell lysates. The yields were ~10.2 and ~2.7 mg per litre of culture respectively. From these results, we concluded that, for proteins that are already stable and soluble when produced with sole poly-histidine tag, thioredoxin does not significantly contribute to the total amount of purified protein. However, for unstable and insoluble proteins such as S15 and S18, the presence of a poly-histidine-thioredoxin tag is crucial for the recovery of recombinant proteins from the soluble fraction (Fig. 3).

In order to improve the solubility of ribosomal protein S15 and S18, the use of a different fusion protein tag, glutathione S-transferase (GST), was also explored. However, expression trials showed that the production of soluble proteins in the cell lysate could not be enhanced (Supplementary Fig. S7), thus confirming the crucial role of thioredoxin. As low temperature may also improve the yield of soluble proteins, further attempts to produce the poly-histidine-thioredoxin- or GST-tagged S15 and S18 by using the *E. coli* ArcticExpress strain were also made. However, we found that the level of soluble proteins in the cell lysate remained low, indicating the use of poly-histidine-thioredoxin tag at ‘high’ temperature (e.g. 18 °C) remains the best strategy to obtaining soluble human ribosomal proteins.

### Separation of fusion tag

In order to obtain pure proteins, proteolytic reaction for the cleavage of thioredoxin fusion tag by TEV protease was tested in different conditions: 4 °C overnight and room temperature (~22 °C) for 3 hours. Interestingly, no cleavage of the thioredoxin tag was observed under the tested condition for S15 and S18. The unsuccessful cleavage could be due to the overall protein conformation, which could obstruct access of the protease to the cleavage site.

In order to create conformational space between the protein-of-interest and the fusion thioredoxin-tag, as well as to facilitate the proteolytic activity of TEV protease, we designed new constructs with three extra glycines immediately after the TEV protease recognition site (and before the ATG start codon). The resulting constructs were cloned into the vector (pNH-TrxT) and transformed into *E. coli* BL21 (DE3) for protein expression. The addition of three flexible amino acids between the fusion tag and the proteins S15 and S18 allowed us to successfully cleave the tags after overnight incubation at 4 °C.

We therefore decided to carry out purification experiments for these L11 and S10 fusion proteins. The separation of the fusion tag from the ribosomal proteins L11 and S10 were achieved by a single step purification using affinity column, as the ribosomal proteins do not have affinity to the column, whilst the TEV protease and the cleaved thioredoxin both contained poly-histidine tags (Supplementary Figs S8 and S9). The purification of S15 and S18 was found to be more challenging. We observed a tendency for these proteins to maintain affinity with other proteins that are present in the column, i.e., they remain bound to the columns when washed with buffers (e.g. from 5 mM to 500 mM of imidazole) that are typically used to remove cleaved proteins from poly-histidine-affinity columns. Interestingly, both S15 and S18 were found to co-elute with the cleaved poly-histidine-tagged TEV protease and poly-histidine-tagged thioredoxin when washed with elution buffer containing 500 mM of imidazole, which is typically used to remove bound fusion tags from poly-histidine-affinity columns. We then tried to break these interactions by washing the column with different buffers. The use of highly basic or highly acidic buffers did not allow the separation of the ribosomal proteins with thioredoxin/TEV protease. Instead, the recovery of pure protein was achieved by washing the column with a buffer containing 6 M guanidine-HCl before elution (Supplementary Figs S10 and S11). Although the presence of guanidine led to the loosening of their tertiary structure, this step turned out to be essential for the recovery of pure S15 and S18 from the affinity column. Pure proteins could then be refolded by washing out the guanidine with a series of spin-concentration and dilution steps. However, whilst S15 could be recovered with good yield, the recovery of S18 turned out to be very low or undetectable in most cases, indicating that the proteins were unstable on their own. In contrast, thioredoxin-tagged S15 and S18 were found to be stable, even when they were left at room temperature for several days. Interestingly, yeast S15 and S18, and their prokaryotic homologous S13p and S19p, were found to be stable as dimers^23,24^, our study thus showed that factors including protein-protein interactions are important for the stability of the proteins in the cytosol and in solution. Unstable human ribosomal proteins are most likely to precipitate if they are separated from their fusion tag. Therefore, it is recommended that these proteins should be studied with their fusion protein-tag attached. Our results also showed that even for proteins that are of similar size and isoelectric point, it is often difficult to find a common optimal condition for their expression and purification.

### Protein characterisation

Biophysical characterisations were then conducted for our recombinant proteins. In order to evaluate if our purified proteins exist as a single or multiple species in solution, pure S10 and L11, and poly-histidine-thioredoxin-tagged S15 and S18, were further purified using gel filtration chromatography. All four chromatograms showed single peaks, indicating that the proteins were present as single species, presumably as monomers (Supplementary Fig. S15). Structural integrity of the purified proteins was evaluated by using circular dichroism (CD) spectroscopy. CD spectra of pure L11 and poly-histidine-thioredoxin-tagged S15 and S18 show secondary structure content, thus indicating a folded state (Supplementary Fig. S16). As a control, we also analysed pure poly-histidine-tagged thioredoxin (Supplementary Fig. S17). The experimental CD measurement of L11 was compared with a simulated CD spectrum generated by using the bioinformatics tool PDB2CD web server. The secondary structural contents appeared to be qualitatively similar between the two spectra. Unfortunately, we could not conduct CD measurements with S10. This is due to the protein’s rapid precipitation in low saline buffers (that are required for CD measurements). The concentration of S10 was too low for a qualitative evaluation.

### Extra copies of tRNAs do not increase translation

In order to improve the yield of the recombinant ribosomal proteins, we analysed the composition of all the four genes (that encode S10, S15, S18 and L11). We found that the gene sequence of most human ribosomal proteins contains codons that are rare in *E. coli*. More specifically, the four genes *RPS10*, *RPS15*, *RPS18* and *RPL11* have similar number of rare codons, respectively 21, 17, 19 and 24 (Table 3). Several studies showed that the expression of genes containing rare codons can be improved using *E. coli* strains which carry extra copies of tRNAs gene in order to provide a higher number of cognate tRNAs, which may increase the production yield of heterologous proteins^25,26^. For this reason, we decided to compare the conventional *E. coli* strains BL21 (DE3) to the strain BL21 (DE3) CodonPlus RIPL (Agilent), which contains extra copies of the tRNA genes *argU* (AGA, AGG), *ileY* (AUA), *proL* (CCC) and *leuW* (CUA). Interestingly, we found that the expression of the three genes *RPS10*, *RPS15* and *RPL11* in the CodonPlus strain led to a decrease in protein production, whilst for *RPS18*, the use of BL21 (DE3) CodonPlus RIPL did not lead to any observable change in yields in all the tested conditions (Fig. 1). Our results are in agreement with studies that showed that the availability of tRNAs did not always correlate with the level of soluble protein produced. In contrast, they may influence translation speed, which may lead to protein misfolding and aggregation^18,27^.

**Table 3:** Rare codons in ribosomal proteins expressed in *E. coli*.

AMINO ACID (CODON)	S10	S15	S18	L11
ARGININE (CGA)	2	0	4	2
ARGININE (CGG)	3	9	3	3
ARGININE (AGG)	1	0	2	2
ARGININE (AGA)	6	0	3	6
GLYCINE (GGA)	3	0	2	2
GLYCINE (GGG)	3	1	2	6
ISOLEUCINE (AUA)	0	0	1	0
LEUCINE (CUA)	1	1	2	1
PROLINE (CCC)	2	5	0	1
THREONINE (ACG)	0	1	0	1
NUMBER OF RARE CODONS (% OF TOTAL CODONS)	21 (12.7%)	17 (11.7%)	19 (12.5%)	24 (13.5%)

## Conclusions

In conclusion, we reported an updated method for the production of human ribosomal proteins in *E. coli* BL21 (DE3), which avoids the recovery from inclusion bodies. The use of a poly-histidine-thioredoxin tag improves the stability and solubility of proteins that cannot be produced in the soluble fraction with the sole poly-histidine tag. It is essential that the induction of heterologous genes takes place at sufficiently low temperatures (e.g. 18 °C or above) in order to restrain the formation of inclusion bodies and avoid aggregation and further precipitation of proteins. It is also important to investigate the appropriate conditions for the induction of protein production, as some proteins can rapidly precipitate whether they are present in high or low concentrations. Nonetheless, this improved method is superior to previous reported methods that require recovery of proteins from inclusion bodies. Thus, we believe our work would facilitate further research into the structure, function and intermolecular interactions of ribosomal proteins

## Methods

### Organisms and growth conditions

*E. coli* strain XL10 Gold, BL21 (DE3),BL21 (DE3) CodonPlus RIPL were grown in LB medium at 18 to 37 °C. *E. coli* ArticExpress was grown in LB medium at 10 to 37 °C. Liquid cultures were incubated with shaking at 150–200 rpm. LB agar was supplemented with 5% (w/v) sucrose for solid cultures. The growth medium was supplemented with appropriate antibiotics.

### Molecular cloning

The cDNAs sequences of the human ribosomal proteins S10, S15, S18 and L11 were purchased from Integrated DNA Technologies. The genes were cloned into the expression vectors pNIC28-BSA4 and pNH-TrxT following the ligation independent cloning protocol described previously^28^. The plasmids pNIC28-Bsa4 and pNH-TrxT were a gift from Opher Gileadi (Addgene plasmid # 26103 and # 26106)^28^. For the production of GST-tagged S18, the vector pGEX-4T-1-S18 was purchased from GenScript. For the production of GST-tagged S15, the synthetic gene was cloned into the expression vector pLJSRSF3 following the protocol described previously^21^. The plasmid pLJSRSF3 was a gift from Hideo Iwai (Addgene plasmid # 64692)^21^.

### Protein expression trials

The synthesis of recombinant proteins was tested in *E. coli* BL21, strains DE3 and Codon Plus RIPL. Overnight cultures were diluted to an OD_600nm_ of 0.05 in a 10 ml culture in falcon tubes, or in a 100 ml culture in Erlenmeyer flasks. When the optical density of the cultures reached approximately 0.5, the expression of the protein was induced by adding different concentrations of isopropyl β-D-thiogalactoside (IPTG) (0, 0.1 and 1 mM) at different temperatures (18 °C to 37 °C). The cells were harvested by centrifugation, and resuspended in a buffer containing 10 mM Imidazole, 500 mM NaCl, 50 mM Tris-HCl pH 7.8, 10% (v/v) glycerol and 2 mM β-mercaptoethanol and lysed by sonication. After another centrifugation at 4 °C for 30 minutes, the cell free extract was analysed and compared to the whole cell sample by SDS-PAGE.

### Protein purification

Five mL of overnight pre-cultures were used to inoculate 500 ml of LB supplemented with kanamycin. Cultures were incubated at 37 °C with 180 rpm shaking. When the OD_600nm_ reached 0.6–0.8, the expression of the gene was induced adding 0.1–1 mM IPTG at 18 °C for about 16 hours. Cells were harvested by centrifugation at 12000 rpm for 30 minutes at 4 °C. Cell pellets were resuspended in a lysis buffer (50 mM Tris-HCl pH 7.8, 500 mM NaCl, 10 mM imidazole, 2 mM Beta-mercaptoethanol, 10% (v/v) glycerol) supplemented with EDTA-free protease inhibitor cocktail (Abcam) with a 1:5 ratio (grams of pellet: mL of buffer). Cells were disrupted by sonication and the lysate was clarified by centrifugation for 30 minutes at 4 °C. Poly-histidine-tagged proteins were purified by affinity chromatography using HisGraviTrap (GE Healthcare). The column was equilibrated with 10 ml of lysis buffer before loading the cell lysate and washed with the same buffer containing 50 mM imidazole. Poly-histidine-tagged proteins were eluted with a buffer containing 500 mM imidazole.

### TEV digestion

One mg of TEV protease was used to digest 10 mg of tagged protein at 4 °C for about 16 hours. After overnight incubation, samples were loaded into a HisGraviTrap column (GE Healthcare) equilibrated with a buffer containing 50 mM Tris-HCl pH 7.8, 500 mM NaCl, 10 mM imidazole, 2 mM β-mercaptoethanol and 10% (v/v) glycerol. A further wash step using a buffer containing 6 M guanidine-HCl was used to recover pure S15 and S18.

### SDS-PAGE protein analysis

Protein analysis was made by using the Bio-Rad Mini-PROTEAN Tetra cell. Precision Plus Protein Kaleidoscope Prestained Standards (Bio-Rad) was used as protein marker. Protein samples were mixed with a buffer containing 50 mM Tris-HCl pH 6.8, 50 mM β-mercaptoethanol, 5 mM EDTA, 2% (w/v) sodium dodecylsulphate, and 10% (v/v) glycerol in a total volume of 20 μL. Samples were heated at 95 °C and electrophoresed on SDS-PAGE at 180 V. After electrophoreses, gels were stained with Comassie blue staining solution and distained in a gentle agitation with a solution of 50% H_2_O, 10% acetic acid and 40% methanol. Pictures of SDS-PAGE gels were taken and cropped using the mobile application Microsoft OneNote for iPhone. No adjustments in colour and contrast were made. Microsoft PowerPoint were used to construct the multi-panel photos and for the addition of arrows and captions.

### Protein analysis by gel filtration analysis and CD spectroscopy

Pure S10 and L11, and thioredoxin-tagged S15 and S18 were analysed by gel filtration and CD spectroscopy. Gel filtration analysis was carried out using HiPrep 16/60 sephacryl S-100 HR (GE Healthcare) with a buffer containing 50 mM Tris-HCl (pH 8), 100 mM KCl, 5% (v/v) glycerol at 0.5 ml/min. 5 to 15 mg of purified protein were injected and 280 nm absorbance was monitored using AKTA Start system (GE Healthcare). CD experiments were performed on a Chirascan spectrometer (Applied photophysics) over the range from 180 nm to 320 nm at 0.5 nm intervals, using a quartz cuvette of 1 mm path length (Hellma). Pure S10, L11 and poly-histidine-tagged thioredoxin, and poly-histidine-thioredoxin-tagged S15 and S18 were concentrated and dialysed a buffer containing in 10 mM sodium phosphate buffer without saline (pH 7.4). All proteins were analysed in a total volume of 350 μL at a concentration of 10 μM exept for S10, which was analysed at a concentration of about 2 μM. Simulated CD spectra of ribosomal protein L11 and thioredoxin were generated using PDB2CD web server^29^.

## Supplementary information

Supplementary Information
