# Supplementary material for: An improved method for the heterologous production of soluble human ribosomal proteins in Escherichia coli
Source: Sci Rep. 2019 Jun 20;9:8884. doi: 10.1038/s41598-019-45323-8 (PMC6586885; doi:10.1038/s41598-019-45323-8)
Supplement: Supplementary file 1 — Supplementary Information [file 41598_2019_45323_MOESM1_ESM.pdf]

SUPPLEMENTARY INFORMATION

An improved method for the heterologous production of soluble human ribosomal proteins in *Escherichia coli*

Danilo Correddu[1], José de Jesús Montaño López[1,2], Praveen G. Vadakkedath[1,3], Amy Lai[1,+], Jane I. Pernes[1,4,+], Paris R. Watson[1,5,+], Ivanhoe K. H. Leung[1,6*]

1. School of Chemical Sciences, The University of Auckland, Private Bag 92019, Victoria Street West, Auckland 1142, New Zealand

2. Facultad de Ingeniería, Universidad Nacional Autónoma de México, Av. Universidad 3000, Ciudad Universitaria, Coyoacán, Cd. Mx., CP 04510, Mexico

3. The MacDiarmid Institute for Advanced Materials and Nanotechnology, Victoria University of Wellington, PO Box 600, Wellington 6140, New Zealand

4. School of Cellular and Molecular Medicine, University of Bristol, Biomedical Sciences Building, University Walk, Bristol BS8 1TD, United Kingdom

5. School of Biological Sciences, Victoria University of Wellington, PO Box 600, Wellington 6140, New Zealand

6. Maurice Wilkins Centre for Molecular Biodiscovery, The University of Auckland, Private Bag 92019, Victoria Street West, Auckland 1142, New Zealand

[+] These authors contributed equally to this work

[*] Correspondence to I.K.H.L. (e-mail: i.leung@auckland.ac.nz)

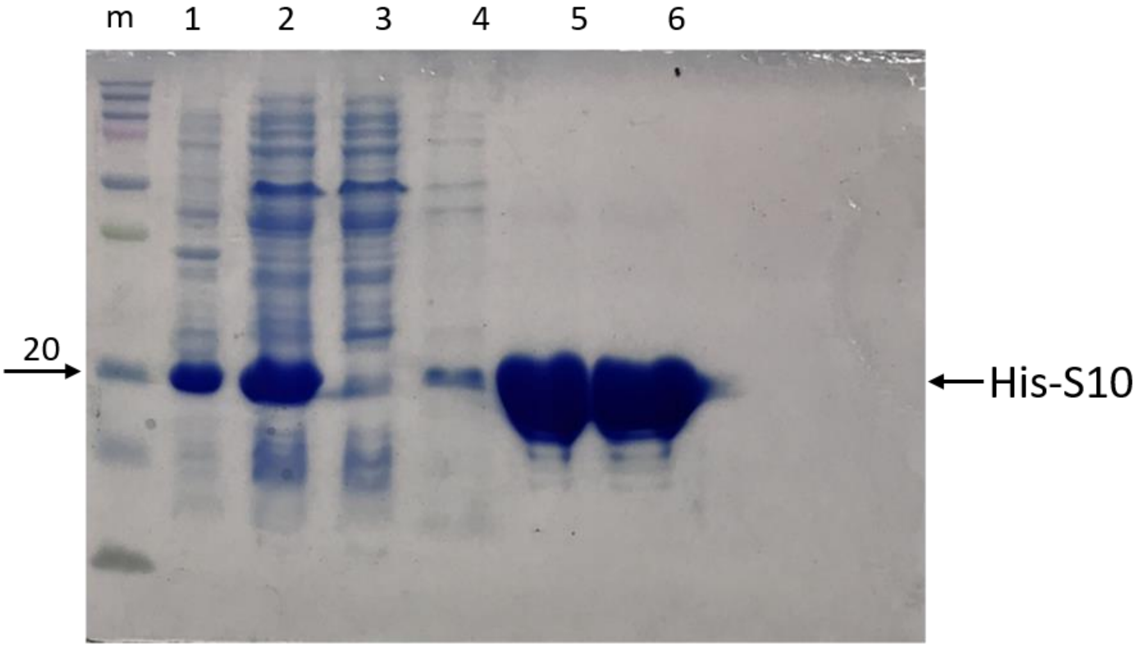

Supplementary Figure S1: SDS-PAGE gel of His-S10 purification. Lanes legend: m= molecular weight protein marker; 1= whole cell sample; 2= cell lysate; 3= flow-through; 4= wash; 5 and 6= elutions. Pure His-tagged ribosomal protein S10 (His-S10) at the expected molecular weight (21.62 kDa) is present in lane 5 and 6 and is indicated by the arrow on the right side of the gel. The picture of the SDS-PAGE gel was taken and cropped using the mobile application Microsoft OneNote for iPhone. No adjustments in colour or contrast were made.

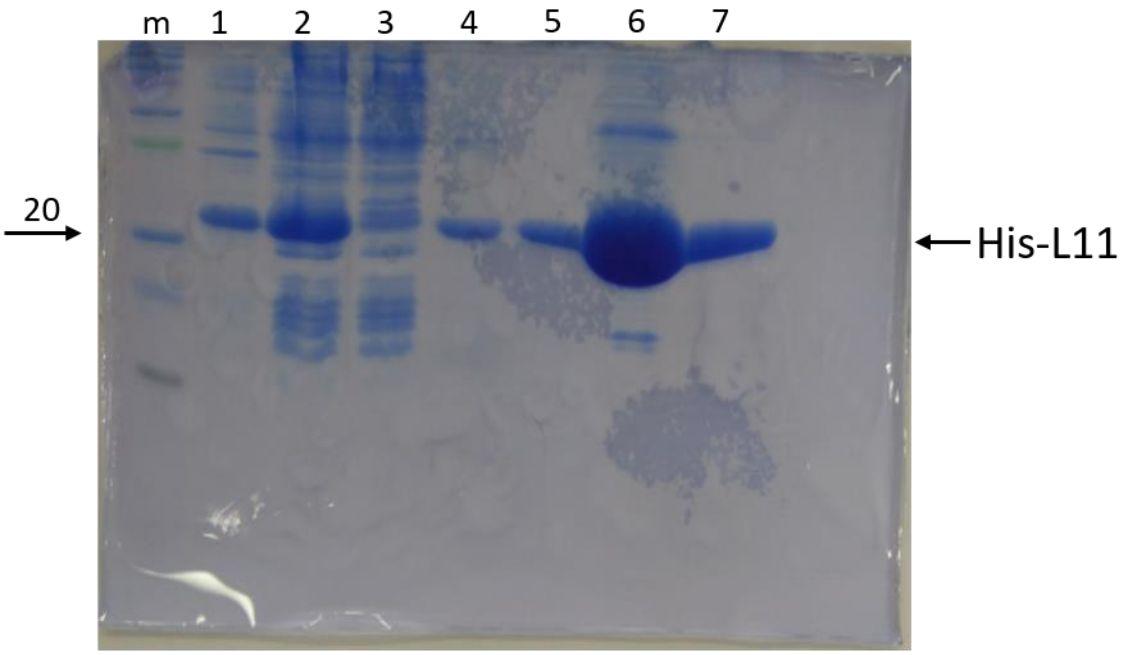

Supplementary Figure S2: SDS-PAGE gel of His-L11 purification. Lanes legend: m= molecular weight protein marker; 1= whole cell sample; 2= cell lysate; 3= flow-through; 4= wash; 5, 6 and 7= elutions. His-tagged ribosomal protein L11 (His-L11) at the expected molecular weight (22.97 kDa) in the elution lanes is indicated by the arrow on the right side of the gel. The picture of the SDS-PAGE gel was taken and cropped using the mobile application Microsoft OneNote for iPhone. No adjustments in colour or contrast were made.

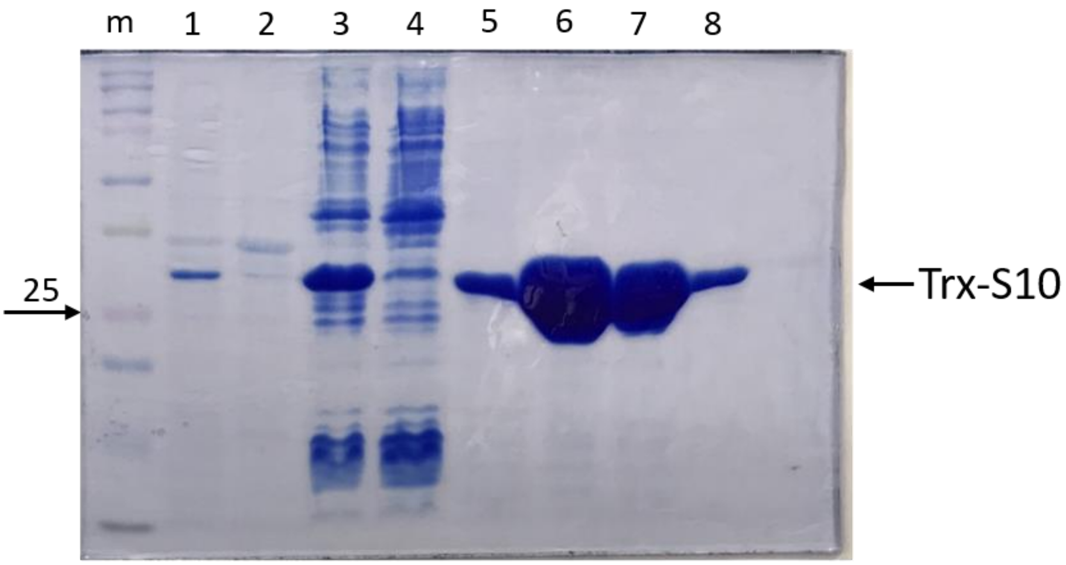

Supplementary Figure S3: SDS-PAGE gel of Trx-S10 purification. Lanes legend: m= molecular weight protein marker; 1= whole cell sample; 2= diluted whole cell sample; 3= cell lysate; 4= flow-through; 5, 6, 7 and 8= elutions. The His-thioredoxin tagged ribosomal protein S10 (Trx-S10) at the expected molecular weight (33.08 kDa) is present in the elution samples (lane 5, 6, 7 and 8) and is indicated by the arrow on the right side of the gel. The picture of the SDS-PAGE gel was taken and cropped using the mobile application Microsoft OneNote for iPhone. No adjustments in colour or contrast were made.

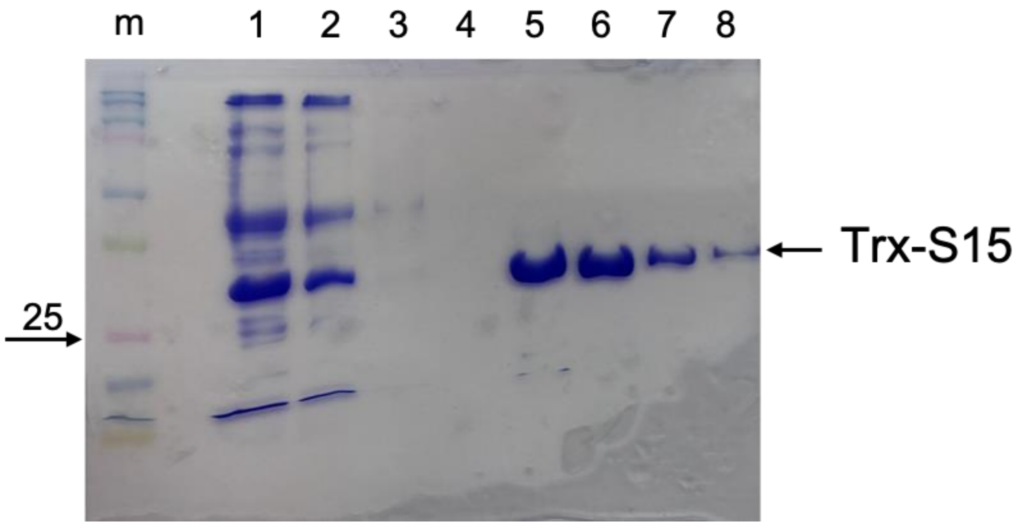

Supplementary Figure S4: SDS-PAGE gel of Trx-S15 purification. Lanes legend: m= molecular weight protein marker; 1= whole cell sample; 2= cell lysate; 3= flow-through; 4= wash; 5, 6, 7 and 8= elutions. Pure His-thioredoxin tagged ribosomal protein S15 (Trx-S15) is present in the elution lanes at the expected molecular weight (31.22 kDa) and is indicated by the arrow on the right side of the gel. The picture of the SDS-PAGE gel was taken and cropped using the mobile application Microsoft OneNote for iPhone. No adjustments in colour or contrast were made.

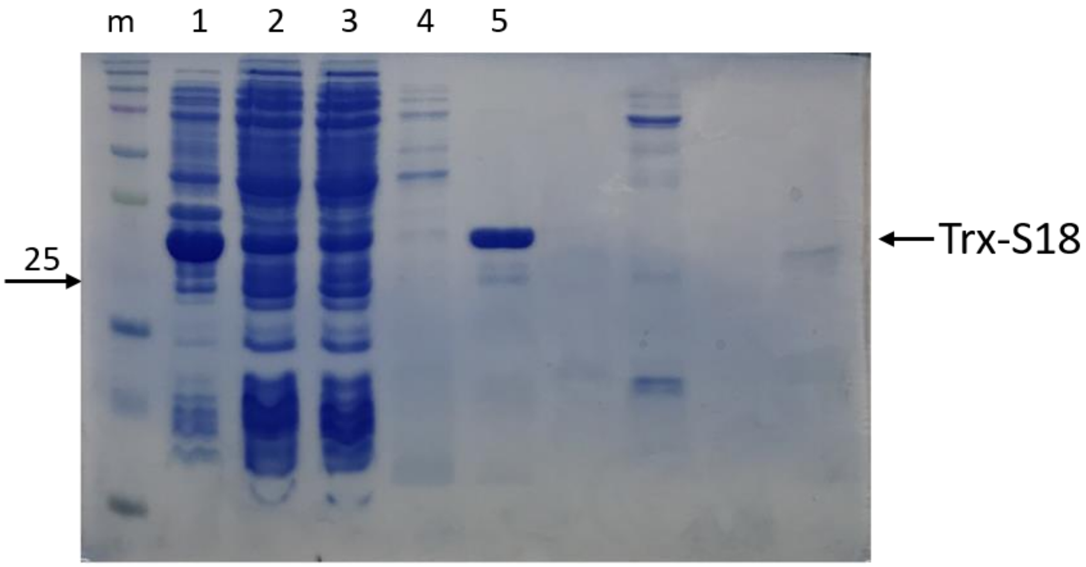

Supplementary Figure S5: SDS-PAGE gel of Trx-S18 purification. Lanes legend: m= molecular weight protein marker; 1= whole cell sample; 2= cell lysate; 3= flow-through; 4= wash; 5= elution. Pure His-thioredoxin tagged ribosomal protein S18 (Trx-S18) is present in the elution at the expected molecular weight (31.73 kDa) and is indicated by the arrow on the right side of the gel. The picture of the SDS-PAGE gel was taken and cropped using the mobile application Microsoft OneNote for iPhone. No adjustments in colour or contrast were made.

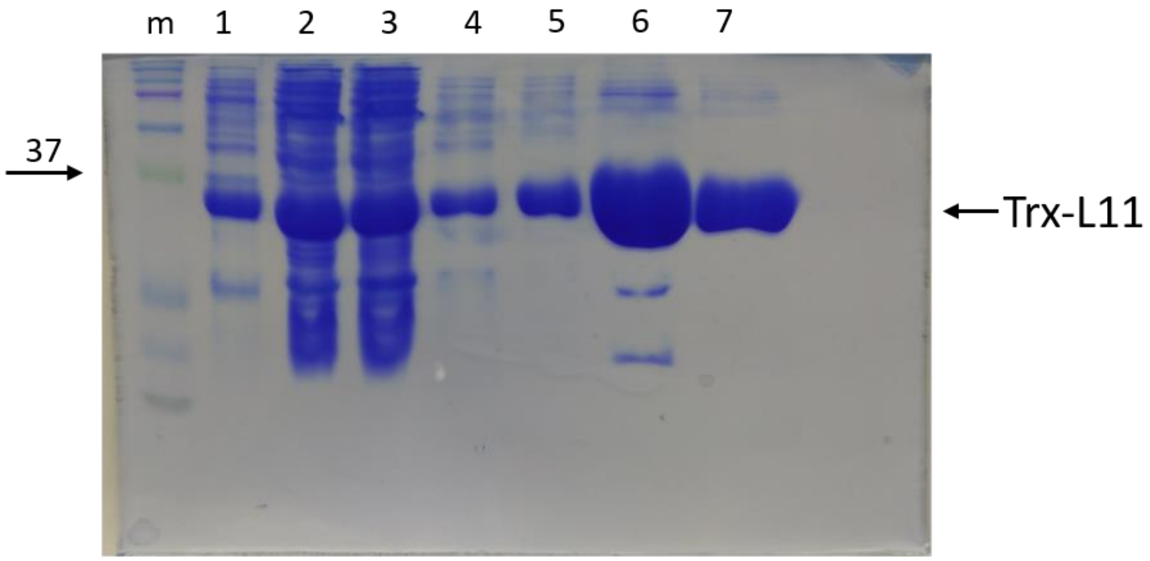

Supplementary Figure S6: SDS-PAGE gel of Trx-L11 purification. Lanes legend: m= molecular weight protein marker; 1= whole cell sample; 2= cell lysate; 3= flow-through; 4= wash; 5, 6 and 7= elutions. His-thioredoxin tagged ribosomal protein L11 (Trx-L11) at the expected molecular weight (34.43 kDa) is present in lane 4, 5, 6 and 7 and is indicated by the arrow on the right side of the gel. The picture of the SDS-PAGE gel was taken and cropped using the mobile application Microsoft OneNote for iPhone. No adjustments in colour or contrast were made.

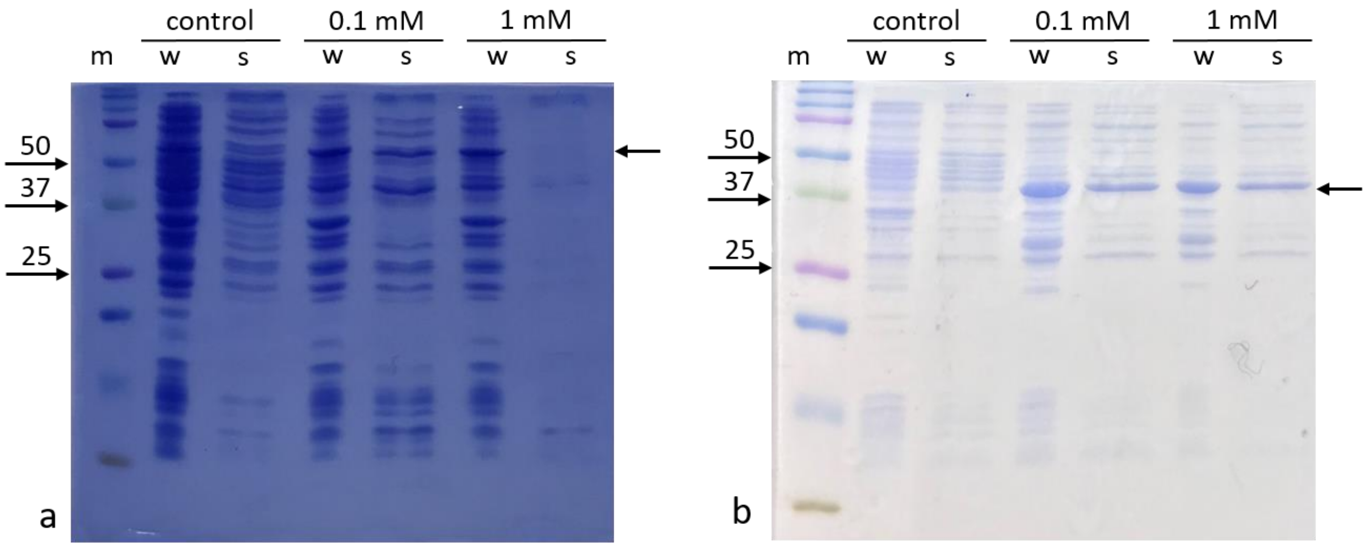

Supplementary Figure S7. SDS-PAGE analysis of protein expression trials in *E. coli* BL21 (DE3) using GST fusion tag. Lanes legend: m= molecular weight protein marker; w= whole cell sample; s= soluble proteins. Every gel includes non-induced samples (control), and samples induced with 0.1 mM and 1 mM IPTG. Gels show expression trials of the GST-tagged ribosomal proteins S15 (a) and S18 (b). Arrows on the right side of the gels indicate the expected positions of proteins (54.81 kDa; 43.86 kDa). Photos of the SDS-PAGE gels were taken and cropped using the mobile application Microsoft OneNote for iPhone. No adjustments in colour or contrast were made.

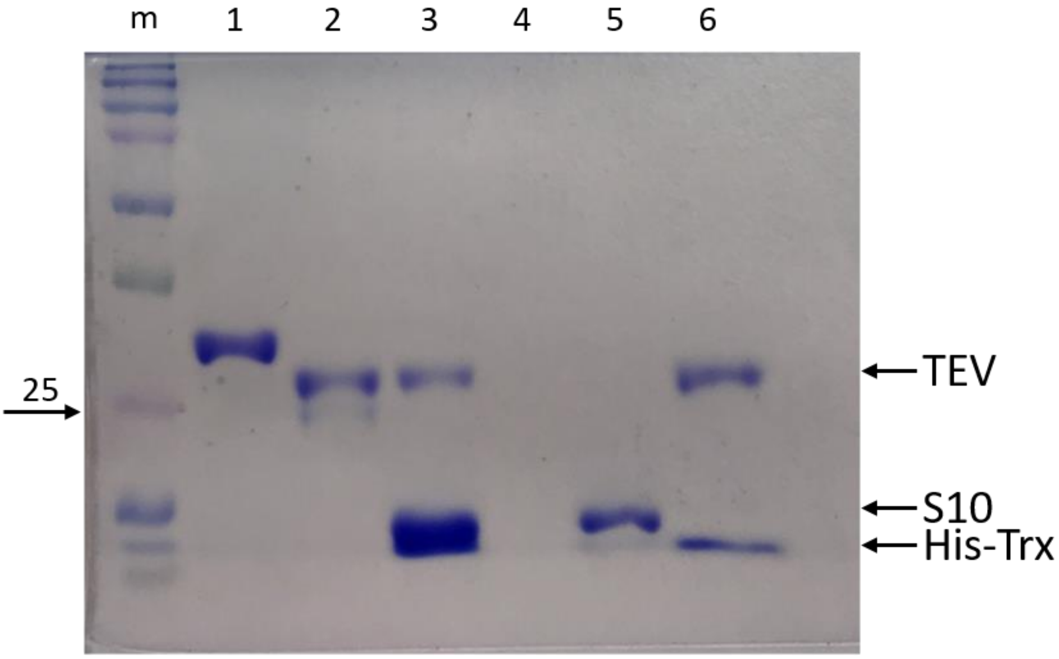

Supplementary Figure S8: SDS-PAGE gel of Trx-S10 digestion with TEV. Lanes legend: m= molecular weight protein marker; 1= His-thioredoxin tagged S10; 2= TEV protease; 3= digestion sample; 4= flow-through; 5= wash, 6= elution. Pure His-thioredoxin tagged ribosomal protein S10 (lane 1) has been digested by TEV protease (lane 2). The digestion sample (lane 3) show two close bands of S10 and His-thioredoxin (His-Trx), respectively at 19.15 and 13.95 kDa, and TEV at 28.12 kDa. Pure S10 is present at 19.15 kDa in lane 5. The picture of the SDS-PAGE gel was taken and cropped using the mobile application Microsoft OneNote for iPhone. No adjustments in colour or contrast were made.

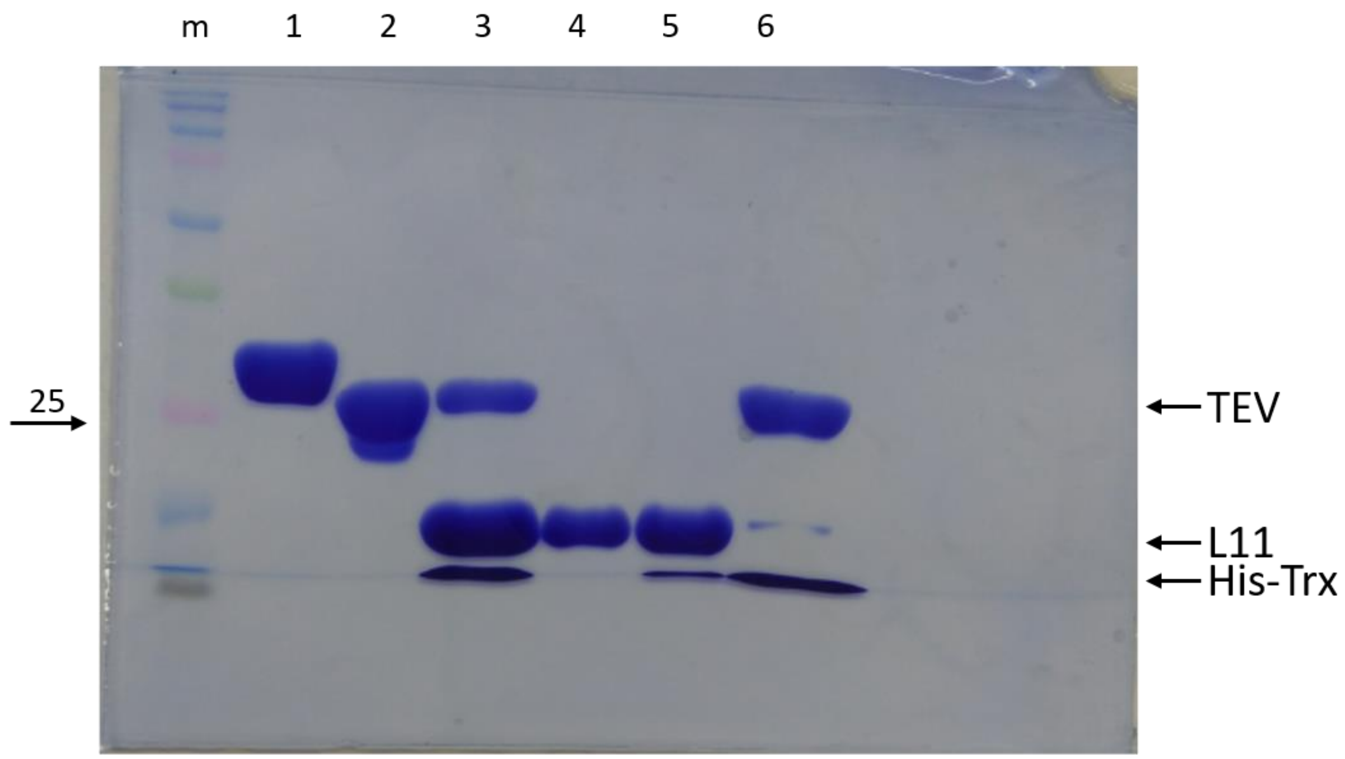

Supplementary Figure S9: SDS-PAGE gel of Trx-L11 digestion with TEV. Lanes legend: m= molecular weight protein marker; 1= His-thioredoxin tagged L11; 2= TEV protease; 3= digestion sample; 4= flow-through; 5= wash, 6= elution. Pure His-thioredoxin tagged ribosomal protein L11 (lane 1) has been digested by TEV protease (lane 2). The digestion sample (lane 3) show three bands, pure L11 and His-thioredoxin (His-Trx), respectively at 20.51 and 13.95 kDa and TEV at 28.12 kDa. Pure L11 is present at 20.51 kDa in lane 4 and 5. The picture of the SDS-PAGE gel was taken and cropped using the mobile application Microsoft OneNote for iPhone. No adjustments in colour or contrast were made.

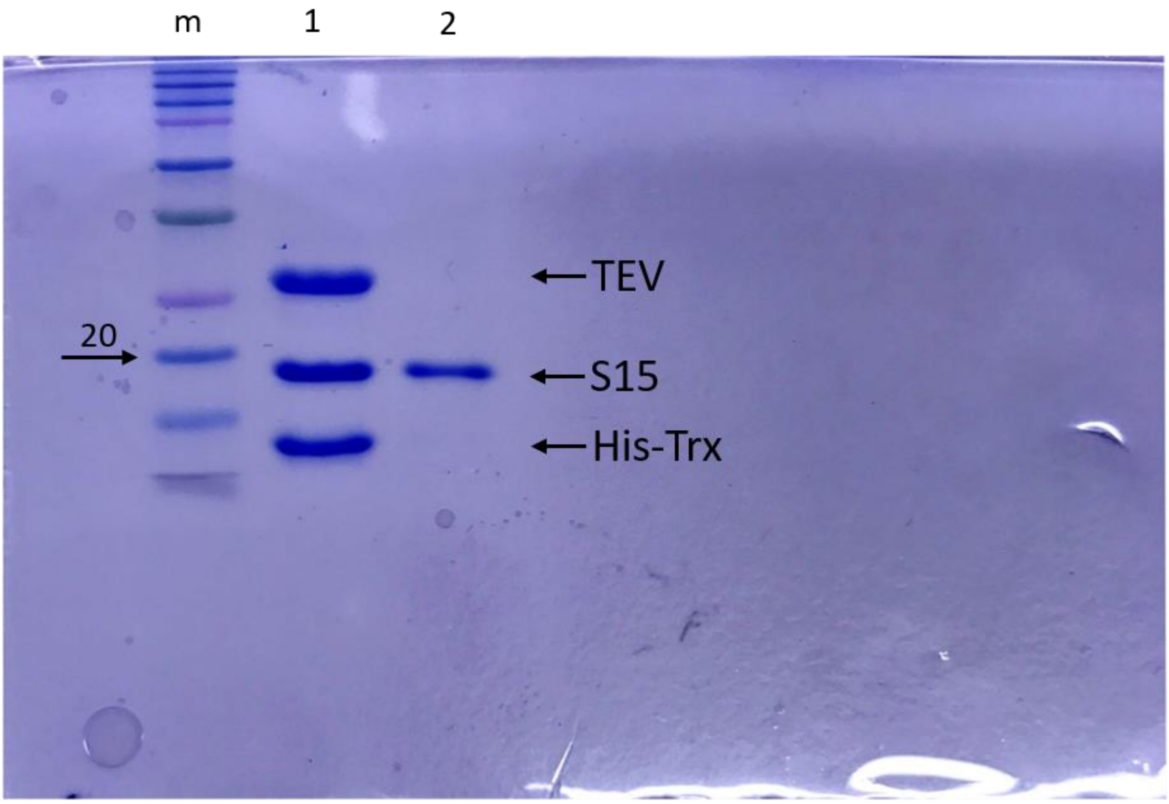

Supplementary Figure S10: SDS-PAGE gel S15 purification. Pure S15 (lane 2) could be recovered from the O/N digestion sample containing a mixture of TEV, S15 and His-thioredixin after a column wash with buffer containing 6 M guanidine-HCl. The picture of the SDS-PAGE gel was taken and cropped using the mobile application Microsoft OneNote for iPhone. No adjustments in colour or contrast were made.

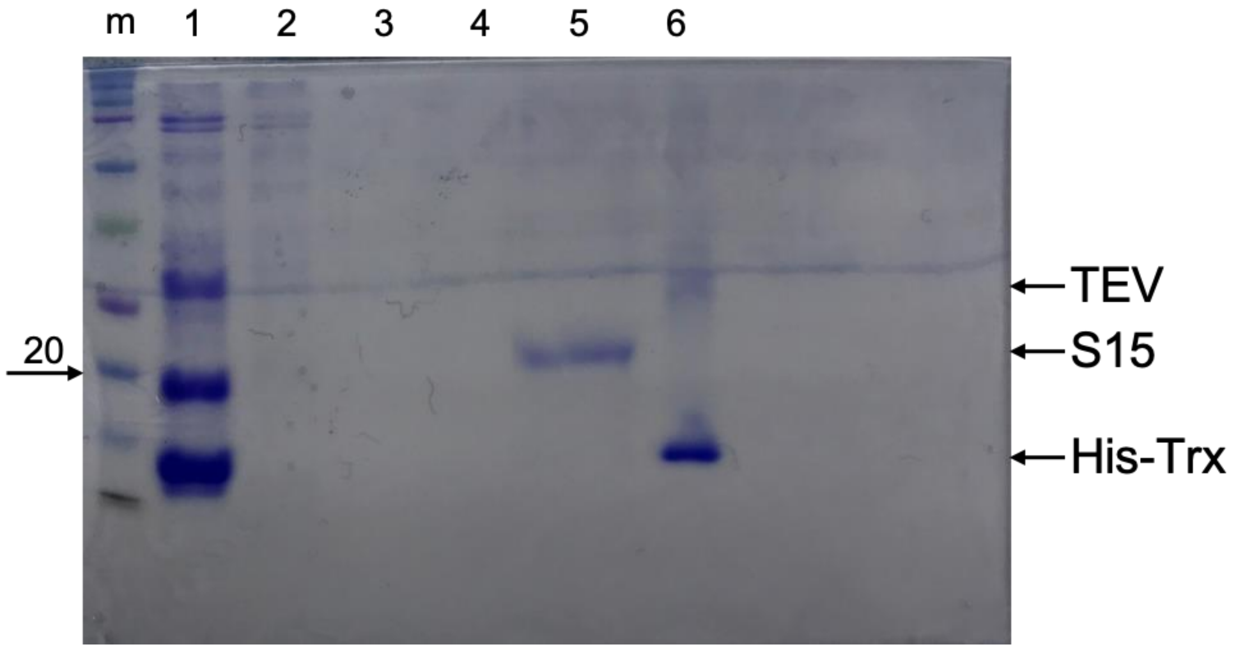

Supplementary Figure S11: SDS-PAGE gel of Trx-S15 digestion with TEV. Lanes legend: m= molecular weight protein marker; 1= digestion sample; 2= flow-through; 3= wash 1; 4= wash 2; 5= wash 3 (containing 6 M guanidine-HCl); 6= elution. Pure His-thioredoxin tagged ribosomal protein S15 (lane 5) could be recovered from the affinity column after a further wash step using 6 M guanidine-HCl. The digestion sample (lane 1) show three bands: TEV, S15 and His-thioredoxin (His-Trx), respectively at 28.12, 17.21 and 13.95 kDa. TEV and His-Trx eluted using elution buffer without guanidine (lane 6). The picture of the SDS-PAGE gel was taken and cropped using the mobile application Microsoft OneNote for iPhone. No adjustments in colour or contrast were made.

RPS10

TACTTCCAATCC
GGCGGCGGC

ATGTTGATGC CTAAGAAGAA CCGGATTGCC ATTTATGAAC TCCTTTTTAA GGAGGGAGTC
ATGGTGGCCA AGAAGGATGT CCACATGCCT AAGCACCCGG AGCTGGCAGA CAAGAATGTG
CCCAACCTTC ATGTCATGAA GGCCATGCAG TCTCTCAAGT CCCGAGGCTA CGTGAAGGAA
CAGTTTGCCT GGAGACATTT CTACTGGTAC CTTACCAATG AGGGTATCCA GTATCTCCGT
GATTACCTTC ATCTGCCCCC GGAGATTGTG CCTGCCACCC TACGCCGTAG CCGTCCAGAG
ACTGGCAGGC CTCGGCCTAA AGGTCTGGAG GGTGAGCGAC CTGCGAGACT CACAAGAGGG
GAAGCTGACA GAGATACCTA CAGACGGAGT GCTGTGCCAC CTGGTGCCGA CAAGAAAGCC
GAGGCTGGGG CTGGGTCAGC AACCGAATTC CAGTTTAGAG GCGGATTTGG TCGTGGACGT
GGTCAGCCAC CTCAGTAA

CAGTAAAGGTGGATA

RPS15

TACTTCCAATCC
(GGCGGCGGC)

ATGGCAGAAG TAGAGCAGAA GAAGAAGCGG ACCTTCCGCA AGTTCACCTA CCGCGGCGTG
GACCTCGACC AGCTGCTGGA CATGTCCTAC GAGCAGCTGA TGCAGCTGTA CAGTGCGCGC
CAGCGGCGGC GGCTGAACCG GGGCCTGCGG CGGAAGCAGC ACTCCCTGCT GAAGCGCCTG
CGCAAGGCCA AGAAGGAGGC GCCGCCCATG GAGAAGCCGG AAGTGGTGAA GACGCACCTG
CGGGACATGA TCATCCTACC CGAGATGGTG GGCAGCATGG TGGGCGTCTA CAACGGCAAG
ACCTTCAACC AGGTGGAGAT CAAGCCCGAG ATGATCGGCC ACTACCTGGG CGAGTTCTCC
ATCACCTACA AGCCCGTAAA GCATGGCCGG CCCGGCATCG GGGCCACCCA CTCCTCCCGC
TTCATCCCTC TCAAGTAA

CAGTAAAGGTGGATA

RPS18

TACTTCCAATCC
(GGCGGCGGC)

ATGTCTCTAG TGATCCCTGA AAAGTTCCAG CATATTTTGC GAGTACTCAA CACCAACATC
GATGGGCGGC GGAAAATAGC CTTTGCCATC ACTGCCATTA AGGGTGTGGG CCGAAGATAT
GCTCATGTGG TGTTGAGGAA AGCAGACATT GACCTCACCA AGAGGGCGGG AGAACTCACT
GAGGATGAGG TGGAACGTGT GATCACCATT ATGCAGAATC CACGCCAGTA CAAGATCCCA
GACTGGTTCT TGAACAGACA GAAGGATGTA AAGGATGGAA AATACAGCCA GGTCCTAGCC
AATGGTCTGG ACAACAAGCT CCGTGAAGAC CTGGAGCGAC TGAAGAAGAT TCGGGCCCAT
AGAGGGCTGC GTCACTTCTG GGGCCTTCGT GTCCGAGGCC AGCACACCAA GACCACTGGC
CGCCGTGGCC GCACCGTGGG TGTGTCCAAG AAGAAATAA

CAGTAAAGGTGGATA

RPL11

TACTTCCAATCC
GGCGGCGGC

ATGGCGCAGG ATCAAGGTGA AAAGGAGAAC CCCATGCGGG AACTTCGCAT CCGCAAACTC
TGTCTCAACA TCTGTGTTGG GGAGAGTGGA GACAGACTGA CGCGAGCAGC CAAGGTGTTG
GAGCAGCTCA CAGGGCAGAC CCCTGTGTTT TCCAAAGCTA GATACACTGT CAGATCCTTT
GGCATCCGGA GAAATGAAAA GATTGCTGTC CACTGCACAG TTCGAGGGGC CAAGGCAGAA
GAAATCTTGG AGAAGGGTCT AAAGGTGCGG GAGTATGAGT TAAGAAAAAA CAACTTCTCA
GATACTGGAA ACTTTGGTTT TGGGATCCAG GAACACATCG ATCTGGGTAT CAAATATGAC
CCAAGCATTG GTATCTACGG CCTGGACTTC TATGTGGTGC TGGGTAGGCC AGGTTTCAGC
ATCGCAGACA AGAAGCGCAG GACAGGCTGC ATTGGGGCCA AACACAGAAT CAGCAAAGAG
GAGGCCATGC GCTGGTTCCA GCAGAAGTAT GATGGGATCA TCCTTCCTGG CAAATAA

CAGTAAAGGTGGATA

Supplementary Figure S12 | Gene sequences: cDNA sequences of the human genes *RPS10*, *RPS15*, *RPS18* and *RPL11*. The four genes contain three extra codons (GGC) before their start codon. Two sequences were added at their 5’ and 3’, TACTTCCAATCC and CAGTAAAGGTGGATA respectively, for the ligation independent cloning protocol described by Gileadi *et al.* (2008).

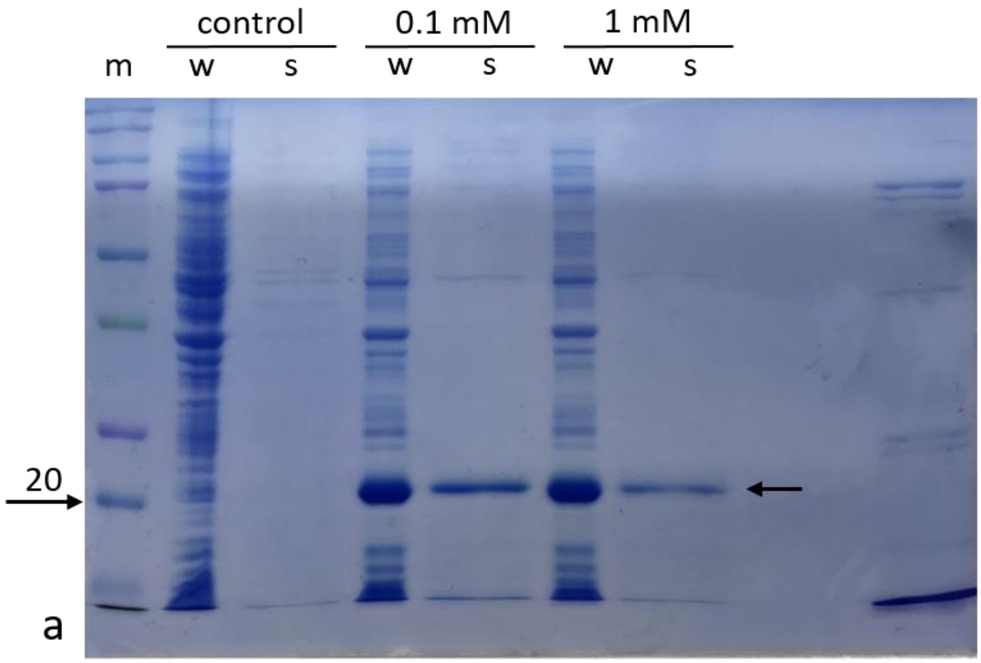
control
0.1 mM
1 mM
m
w
s
w
s
w
s
20
a

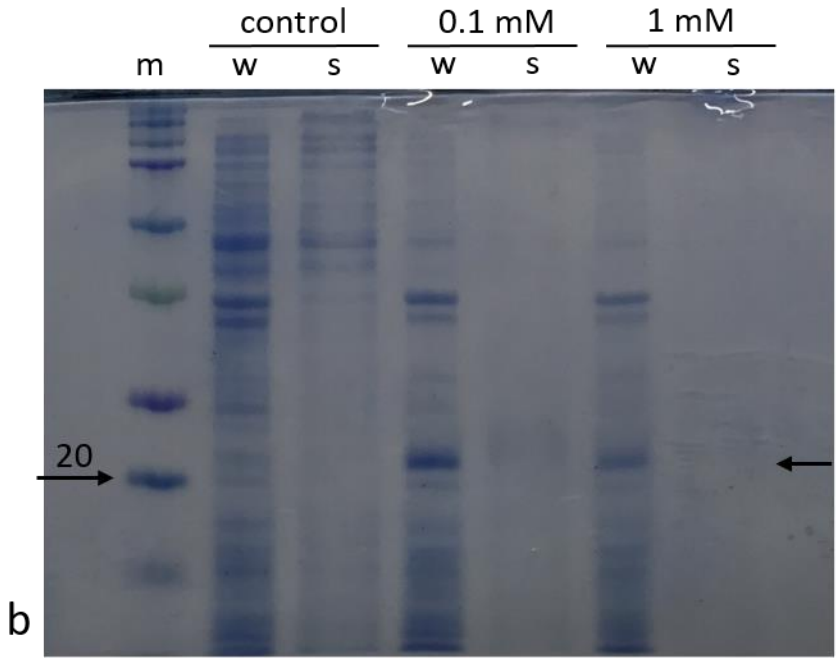
control
0.1 mM
1 mM
m
w
s
w
s
w
s
20
b

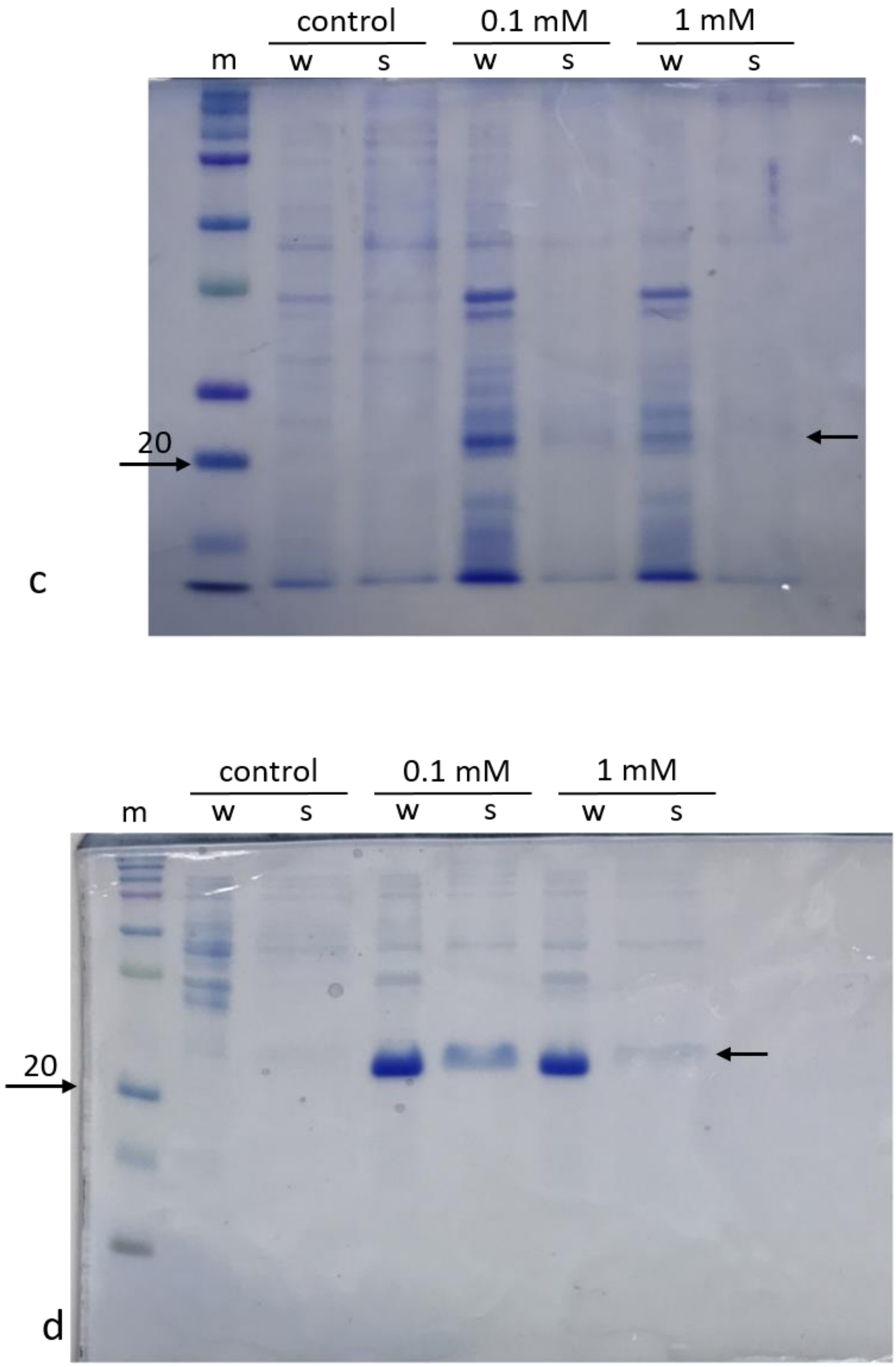

Supplementary Figure S13: Full-length SDS-PAGE gels from main text Figure 2.

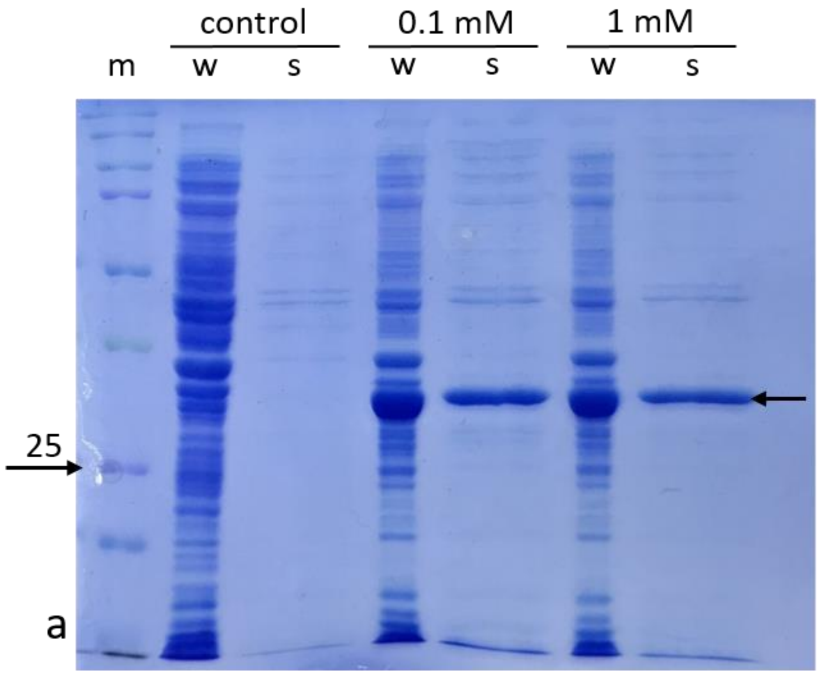
control
0.1 mM
1 mM
m
w
s
w
s
w
s
25
a

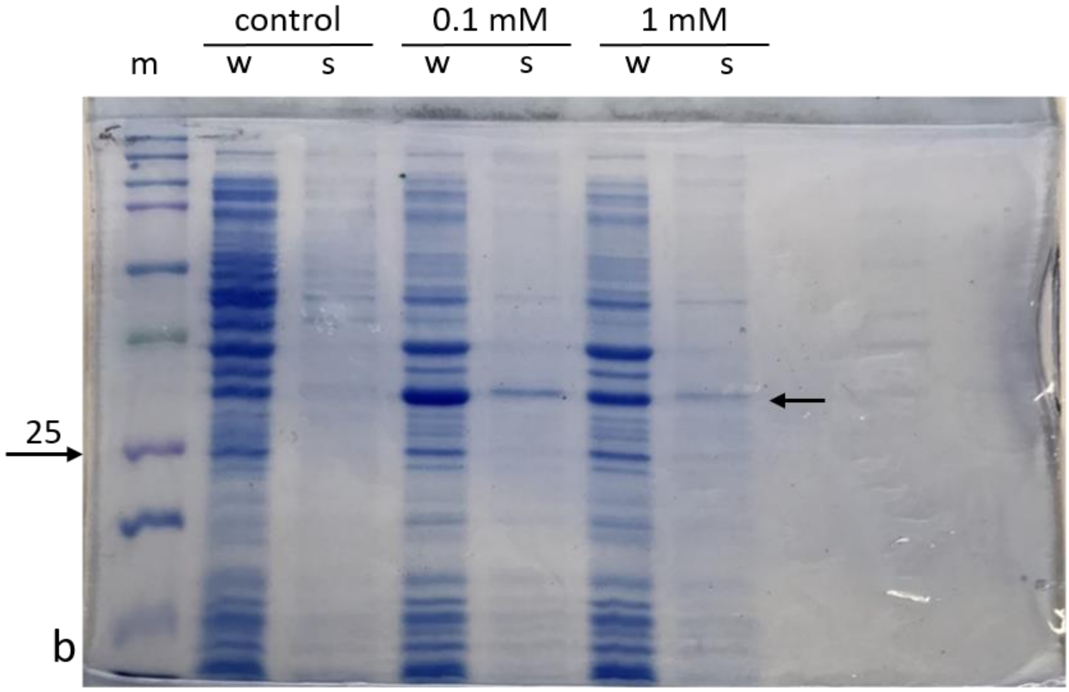
control
0.1 mM
1 mM
m
w
s
w
s
w
s
25
b

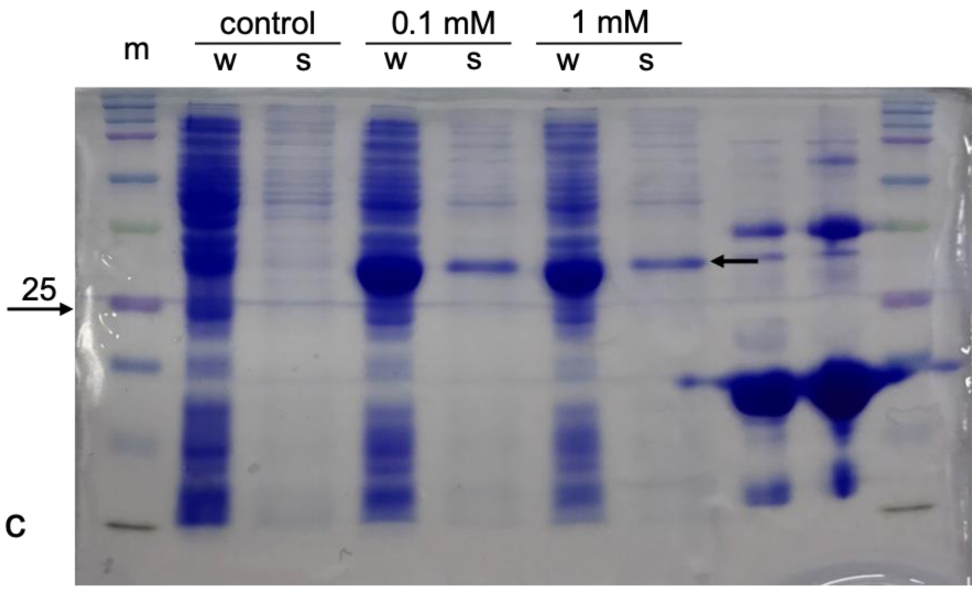

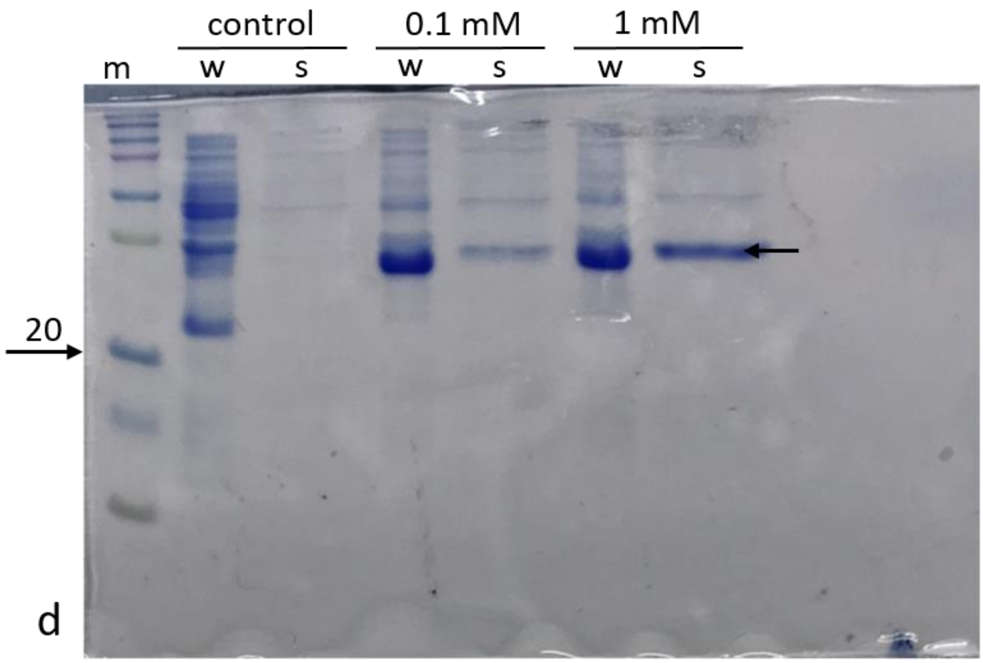

Supplementary Figure S14: Full-length SDS-PAGE gels from main text Figure 3.

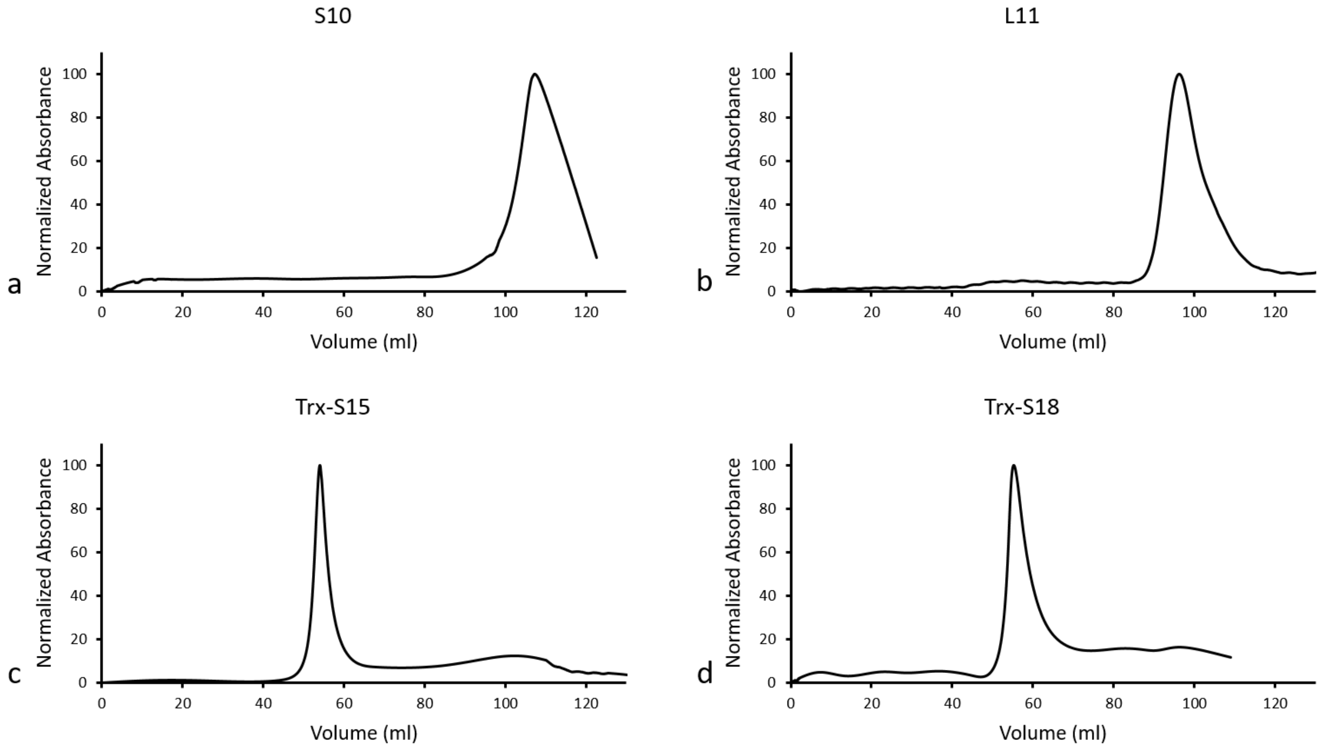

Supplementary Figure S15: Gel filtration chromatograms of pure ribosomal protein S10 (a) and L11 (b), and thioredoxin-tagged S15 (c) and S18 (d).

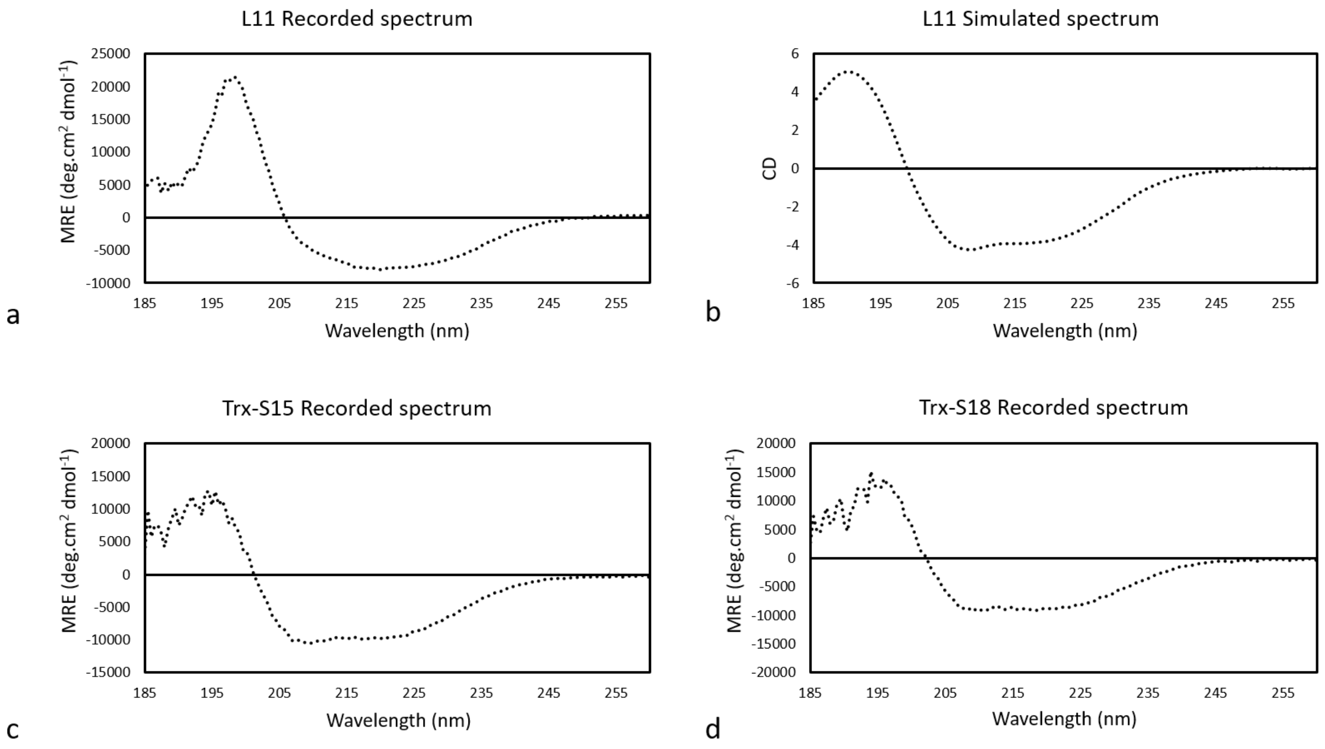

Supplementary Figure S16: Experimentally recorded CD spectrum of 10 μM of the ribosomal protein L11 (a) and its simulated spectrum generated with the bioinformatics tool PDB2CD web server (b); experimentally recorded CD spectrum 10 μM of the thioredoxin-tagged S15 (c) and thioredoxin-tagged S18 (d).

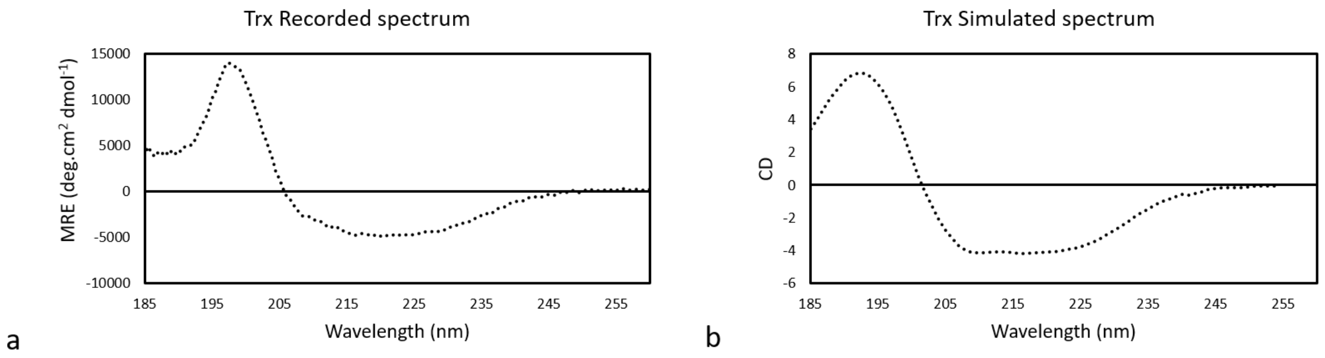

Supplementary Figure S17: Experimentally recorded CD spectrum of 10 μM of his-tagged thioredoxin (a) and its simulated spectrum generated with the bioinformatics tool PDB2CD web server (b).
